# Sequential Injection System for Analysis of Degree Brix, Orthophosphate and pH in Raw Sugarcane Juice Applicable to Sugar Industry

**DOI:** 10.3390/molecules26216484

**Published:** 2021-10-27

**Authors:** Phoonthawee Saetear, Nattinee Saechua, Kamonthip Sereenonchai

**Affiliations:** 1 Flow Innovation-Research for Science and Technology Laboratories (Firstlabs), Mahidol University, Rama 6 Road, Bangkok 10400, Thailand; nattinee@outlook.com; 2 Department of Chemistry and Center of Excellence for Innovation in Chemistry, Faculty of Science, Mahidol University, Bangkok 10400, Thailand; 3 Department of Chemistry, Faculty of Science and Technology, Thammasat University, Pathum Thani 12120, Thailand

**Keywords:** sugarcane juice, pH, Brix, phosphate, schlieren effect, molybdenum blue, ISFET, sequential injection

## Abstract

This work presents, for the first time, a new sequential injection analysis (SIA) method to simultaneously analyze degree Brix, orthophosphate and pH in raw cane juice. These key parameters relate to price of harvested sugarcane and quality of cane juice for sugar production. The SIA system employed two detectors: the first detector is a diode-array spectrophotometer, equipped with a regular flow cell, for measurements of degree Brix and orthophosphate. Quantitative of degree Brix (°Bx; ca. % (*w*/*w*) sucrose) was based on manipulation of the schlieren effect at the interface between plugs of sample and water. Orthophosphate analysis was carried out based on the molybdenum blue method with significant reduction in consumption of the reagents. Compensation of the schlieren effect from sucrose for determination of orthophosphate was achieved by using a dual-wavelength spectrometric detection. Second detector is a pH-sensing device, called ion-selective field-effect transistors (ISFET). The ISFET is based on the current through the ISFET arising according to the H^+^ concentration in solution. Our developed SIA system provides linear calibration graphs fitting for purpose in analysis of sugarcane juice (pH: 0–14, °Bx: 1.0–7.0 and P_2_O_5_: 20–200 mg L^−1^). Simultaneous analysis of sugarcane juice for pH, °Bx and P_2_O_5_ is carried out within 5 min (12 sample per h). Precision of SIA system is acceptable (RSD < 3%). Our SIA system gave quantitative results insignificantly different, as compared with conventional methods for analysis of pH, °Bx and P_2_O_5_ in sugarcane juice.

## 1. Introduction

The price of harvested cane is based on the sugar content or degree Brix (°Bx) in the raw cane juice; the higher the sugar content, the higher the price of the cane stalks. In Thailand, °Bx is the sugar content of an aqueous solution. One degree Brix is defined as 1 g of sucrose in 100 g of solution. If the solution contains dissolved solids other than pure sucrose, then the °Bx only approximates the dissolved solid content. Normally, the content of sugar found in raw cane juice is between 8 and 16 °Bx [1,2]. In the cane sugar industry, °Bx is measured before delivery to the mill. The most commonly used method for determination of °Bx in raw cane juice is polarimetry [1]. The raw cane juice needs to be clarified with lead acetate for precipitating fibers and impurities, and then filtered through a cellulose membrane prior to analysis. These steps require about 20 min before the clarified juice is ready for measurement.

Several methods for the determination of sucrose in cane juice have been proposed. Liquid chromatography with differential refractometry detection has been applied for saccharides, including sucrose in sugar cane, using an ion-exchange column [3,4]. An attenuated total reflectance Fourier transform infrared spectrometry, with multivariate analysis [5], has been employed to measure sucrose content in cane juice using the characteristic absorption bands of sucrose at 800–1250 cm^−1^. An X-ray spectrometry and multivariate analysis [6] has also been reported for direct determination of sugar can quality parameter, including sucrose and fiber contents. Thermometry has been reported by Thavarangkul based on the hydrolysis of sucrose by invertase enzyme immobilized on a sensing thermistor [7]. The heat generated during the hydrolysis reaction of sucrose was measured from the temperature difference between reference and sensing thermistors.

For automated system, flow injection (FI) technique has also been employed. Zagatto and his group have reported a spectrophotometric-FI method for quantitative analysis of sucrose and total reducing sugars [8,9]. Another spectrophotometric-FI method was also proposed for determination of total reducing sugars using a focalized coiled reactor in a microwave oven in order to improve the sensitivity [10]. A gravimetric-FI method has also been developed by weighing on-line precipitates of Cu_2_O produced by Fehling reagent [11]. Accumulation of Cu_2_O on a filtration unit fitted to the underside of the plate of an analytical balance was continuously monitored. A group of researchers reported a piezoelectric-FI method for analysis of cane juice [12]. Sucrose content changed the frequency of the 10 MHz piezoelectric quartz crystal due to variation of sample viscosity and density. In addition, a multi-commutation flow-based approach [13] was also developed for glucose analysis employing enzymatic reaction with glucose-oxidase.

After evaluating the price of the sugar cane, the stalks are conveyed for chopping and crushing to obtain the raw sugarcane juice. This solution is yellow and cloudy due to tissue debris and suspension of particles of soil and dust. Clarification of this turbid sugarcane juice is required prior to further purification process for production of the sugar syrup. In the clarification process, ‘milk of lime’ or calcium hydroxide [14] is added to the liquid to give calcium phosphate precipitates. The calcium phosphate precipitates remove suspended particles, as well as adsorbing the colored compounds. The raw sugarcane juice contains phosphate in the form of orthophosphate PO_4_^3−^, and determination of this phosphate content is required prior to the clarification step. In the sugar industry the content of phosphate in raw crushed juice is reported in terms of P_2_O_5_ content due to the standard classical method that has an ashing step, which oxidizes the orthophosphate to P_2_O_5_.

In the literature [14], the recommended level of phosphate in cane juice is between 300–400 mg P_2_O_5_ L^−1^. Low phosphate contents lead to inefficient clarification and must be supplemented with orthophosphoric acid. However, too high phosphate content can cause difficulty in the filtration step and scaling in the evaporator boiler [14]. Thus, it is necessary to measure the phosphate content of the sugarcane juice to ascertain the optimal economic condition for clarification. The common method of analysis is the colorimetric molybdenum blue method. Reducing agents employed in the molybdenum blue method include ascorbic acid, stannous chloride, 1-amino-2-naphthol-4-sulfuric acid and sodium sulfide [15,16]. Ion chromatography (IC) has also been employed to eliminate interferences from silicate ion and the background color of cane juice [17].

Besides °Bx and orthophosphate analysis, pH monitoring is also a key parameter for sugar production. The pH of the raw sugarcane juice is initially low at approximately 4.5–5.5. Addition of milk of lime in the clarification process can initially bring the pH up to 7–9, causing decay of sucrose to fructose and glucose. Eventually, liming increases the pH up to 11.0–11.5 [18]. During the processes of sugar production, pH is therefore crucial and is required for continuous pH monitoring to ensure the complete and efficient processing.

According to the literature background, degree Brix (as sucrose), orthophosphate and pH are key parameters for the economic production of sugar. To the best of our knowledges, there has no report of flow-based method for the simultaneous analysis of sucrose, orthophosphate and pH in sugarcane juice. Our group therefore developed a sequential injection analysis (SIA) module incorporated with two detectors for three key parameters. Sucrose (equivalent to °Bx) and orthophosphate was carried out using a benchtop spectrophotometer. Sucrose was detected based on the schlieren effect at the interface between sample and pure water plugs [19,20,21,22,23,24], as our reported for analysis of sucrose in beverages [19,20,21]. Orthophosphate analysis was achieved by the molybdenum blue method, instead of turbidimetry [19], to avoid the adsorption of the in-line precipitates on the inner wall of PTFE tubing. Dual-wavelength spectrophotometric detection [25] was exploited to compensate the schlieren effect contributed from sucrose. Measurement of pH was carried out based on a pH-sensing device, called ion-selective field-effect transistors (ISFET). The ISFET is based on the current through the ISFET arising accordingly to the H^+^ concentration in solution. The developed SIA system was applied to raw sugarcane juice as compared to comparative methods. Our proposed SIA system is suitable for monitoring these key parameters during the entire sugar processing, from price evaluation to sugar refinement.

## 2. Results and Discussion

### 2.1. System Design

**Figure 1:**
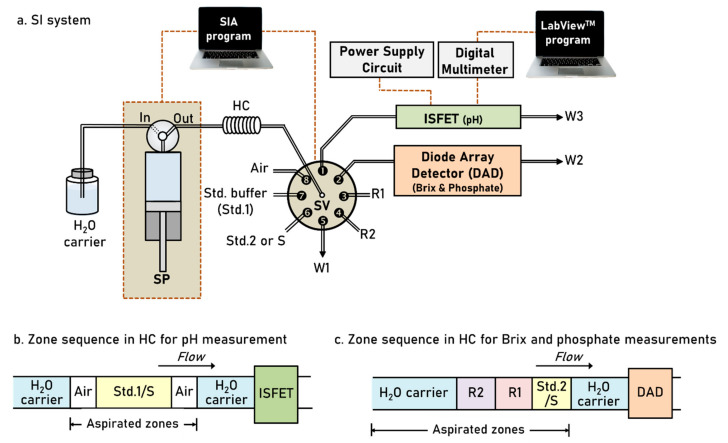
Schematic diagram of (**a**) the SIA system with zone sequences for measurement of (**b**) pH and (**c**) Brix and orthophosphate in sugarcane juice. SP: syringe pump; SV: 8-port selection valve; HC: holding coil; Std.1: standard buffer solution (pH 4.01, 7.00, 10.01); Std.2: standard mixture of sucrose and phosphate; S: sugarcane juice sample; R1: 9.7 mmol L^−1^ molybdate in 0.6 mol L^−1^ sulfuric acid; R2: 0.2 mol L^−1^ ascorbic acid; W1–W3: wastes; ISFET: ion-selective field effect transistor; DAD: photodiode array detector.

**Figure 2:**
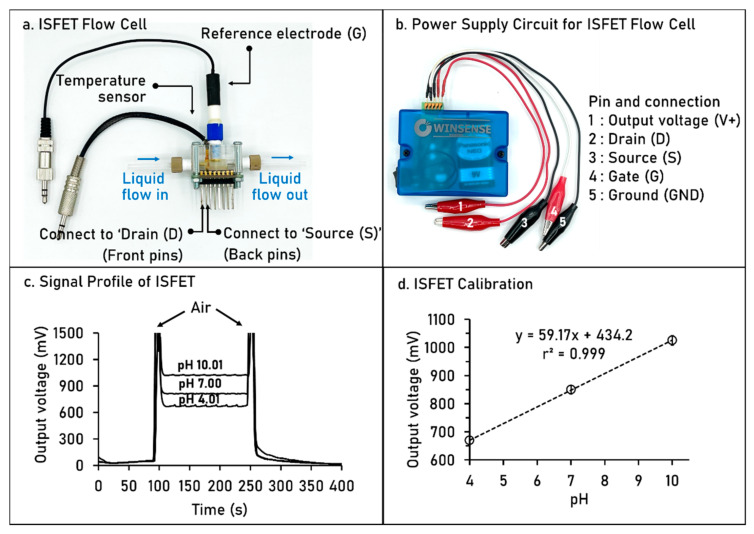
Images of (**a**) the ISFET flow cell, with the liquid flow lines (in and out), connected to (**b**) power supply circuit, for measuring the pH responses showing as (**c**) recorded signal profile and (**d**) corresponding calibration graph obtained from standard buffer solutions pH 4.01, 7.00 and 10.01.

The SIA system, shown in Figure 1, was used to develop a method for simultaneous analysis of pH, degree Brix (°Bx) and orthophosphate in sugarcane juice. The SIA system was in-line coupled with two detectors: ISFET flow-through cell (Figure 2a) connected to a power supply circuit (Figure 2b) and photodiode array detector (DAD) for analysis of pH and °Bx and orthophosphate, respectively. Prior operation of analysis cycle, the system fills each discrete flow line (connected to SV) with standards and reagents as well as a main flow line to detectors with DI water.

The system starts by aspirating the detection zone into holding coil (HC) for pH measurement (see Figure 1b). Air plugs were used to sandwich the standard buffer (Std.1) or sample (S) for avoiding the dilution along the flow line via port 1 of selection valve (SV). Standard buffer or sample solution was then kept in the ISFET flow cell with contacting to ISFET and reference electrodes. Output signal as voltage was recorded (Figure 2c) to construct the calibration curve (Figure 2d) for pH measurement.

During standard buffer or sample solution held in the ISFET flow cell, the system draws DI water to clean the main flow line of HC, via port 5 of SV, to ensure non-contamination for measurements of °Bx and orthophosphate. Detection zone in Figure 1c was then aspirated in HC. A mixed standard (Std.2) of sucrose and phosphate or sample (S) was drawn as the last plug of the detection zone as a smooth and sharp interface, with a typical concentration gradient, at zone head (S/H_2_O, see Figure 1c) is required to detect the light refraction (schlieren effect) for °Bx analysis. Orthophosphate analysis was carried out by detection of light absorption of molybdenum blue complex at the zone tail (R2/R1/S), see zones in Figure 1c. Dual-wavelength spectrophotometry for °Bx and orthophosphate analysis was given in details in Section 2.3 and Section 2.4.

### 2.2. ISFET Flow Cell for pH Measurement

Ion-selective field-effect transistor (ISFET) is one type of the field effect transistors. The ISFET, conventionally referred to a pH sensor, has been employed based on potentiometric measurement of concentration of H^+^ or OH^−^ ions in solution. Briefly, the ISFET consists of three terminals: the source (S), drain (D) and gate (G) (see Figure 2a). The ISFET’s source and drain are constructed as for a metal-oxide semiconductor field-effect transistor (MOSFET), but with the metal gate replaced by an ion-selective membrane, measuring electrolyte solution and reference electrode. The gate electrode is separated from the channel by a barrier which is sensitive to H^+^ ions and a gap to allow the measuring solution under test to approach in contact with the sensitive barrier. An ISFET’s threshold voltage depends on the pH of the measuring solution in contact with its ion-sensitive barrier. The voltage between the source and drain of the ISFET regulates the current flow in the gate voltage. The ISFET is therefore based on the current through the ISFET arising accordingly to the H^+^ concentration in solution, used as the gate electrode. An output voltage between source and drain was then measured.

Regarding the SIA flow system, an ISFET flow cell (Figure 2a) was incorporated as detection cell for pH measurement. To operate the ISFET, power supply circuit with battery (Figure 2b) was connected to the ISFET flow cell via crocodile clips 2, 3 and 4 to pins of drain (D), source (S) and gate (G), respectively, in ISFET flow cell Figure 2a. The output signal was recorded with connection of crocodile clips 1 and 5 to a digital multimeter. With operational step in Table 1, the signal profile of pH measurement was obtained as shown in Figure 2c. The calibration process is similar to conventional pH glass electrode and pH meter in which plotting the output voltage (y-axis) vs. pH of standard buffer solutions (x-axis). We designed the aspirated plug of buffer solution sandwiched with air segment in order to avoid the dilution effect along the flow path with water carrier, resulting in observation of spike signal of air (see Figure 2c). Calibration plot was then obtained as shown in Figure 2d with slope value closed to Nernst’s equation. In this work, we also separate the liquid-flow path for pH measurement as depicted in zone sequence in Figure 1b.

### 2.3. The Schlieren Effect for Brix Analysis

In flow injection analysis (FIA) and its related techniques, the schlieren effect is observed when injecting a medium with high refractive index (RI) into a medium with lower RI, or vice versa. Such actions result in a concentration gradient that can cause artefact signal with optical detectors due to the light deflection (reflection and refraction) as it passes through this gradient [22,25,26]. The schlieren effect in FIA was first mentioned by Krug et al. in 1977 [24]. Nonetheless, the schlieren effect can be useful in flow-based analysis [19,20,21]. Herein, we manipulate a positive aspect of the ‘schlieren effect’ (sometimes called as ’refractive index effect’ or ‘lens effect’), as an in-line detection method for °Bx analysis in raw sugarcane juice.

Our group reported the use of Schlieren effect with SIA-based operation, implemented with a paired emitter–detector diode (PEDD) sensor for analysis of sucrose in cola drinks. The analytical signals for sucrose were well-defined and reproducible. However, the source of the generated schlieren signals has not been mentioned. In this work, we still employed SIA as operational system with addition of discussing the source of generated schlieren signals and the geometry of the schlieren signal, which was obtained from the photodiode array spectrophotometer.

**Figure 3:**
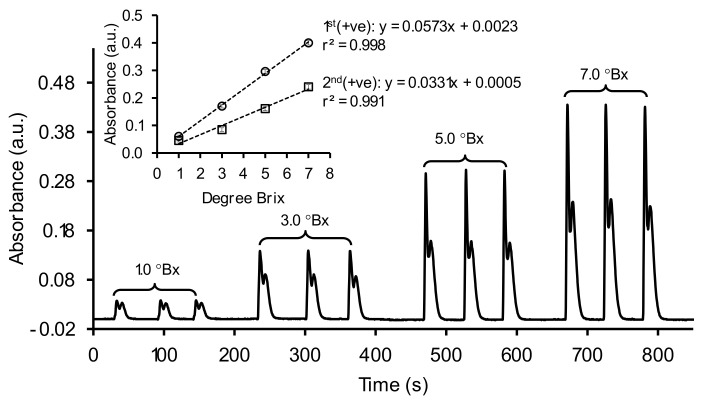
The Schlieren signal obtained from various standard sucrose solutions (1.0 to 7.0 °Bx) under the detection wavelength of 1000 nm. Inset: linear calibration graphs obtained from data acquisition with two positive peaks.

We initially employed the SIA system (Figure 1a) to aspirate a single plug of sample (S) as the detection zone, as almost similar to zone sequence shown in Figure 1c without R1 and R2. With using standard sucrose solutions (1.0 to 7.0 °Bx), the signal profiles in Figure 3 show two positive (+ve) signals: 1st (+ve) and 2nd (+ve) signals, representing the liquid boundary for zone head (S/H_2_O) and zone tail (H_2_O/S), respectively. The profiles were similar to those from previous report [21], except for 1.0 °Bx, due to differences in optical alignment and type of light detector. However, the schlieren signal were well-defined and reproducible for triplicate injections (see Figure 3). Linear calibration graphs (r^2^ > 0.990) were obtained by plotting either the 1st (+ve) or the 2nd (+ve) peaks as analytical signals in values of absorbance (a.u.: arbitrary unit). For application to sugarcane juice sample, the calibration plot from the 1st (+ve) signal was used due to its higher sensitivity (slope of calibration plot).

Regarding the high repeatability of the schlieren signals, it indicates that the SIA system was under good mixing condition with establishment of a relatively steady liquid lenses [22,27]. As our flow operation under laminar flow condition, isolines of different concentrations, and thus refractive index (RI) acting as lenses (*n*_water_: 1.3330, *n*_sucrose (1_._0–7_._0 °Bx)_: 1.3344 to 1.3433; *n* = RI), are established along the flowing sample. Precise measurement is then affected to a lesser extent and the intensity of the schlieren signal is proportional to the concentration of sucrose. In this situation, characteristic of the Schlieren signals should be considered as a blank, not a noise, as they are concentration dependence (see calibration graphs in the inset of Figure 3). Therefore, the Schlieren effect is able to be manipulated for quantitative analysis of known target samples.

### 2.4. Compensation of Schlieren Effect for Orthophosphate Analysis

With aspiration of zone sequence in Figure 1c, plugs of the reagents (R1 and R2) for orthophosphate analysis were first aspirated through the ports 4 and 5 of SV into coil HC (Figure 1a), followed by aspiration of plug S, which was either pure water, 3 °Bx sucrose, 100 mg P_2_O_5_ L^−1^ or a solution of 3 °Bx sucrose with 100 mg P_2_O_5_ L^−1^. This sequence, H_2_O/R2/R1/S/H_2_O, were then pushed in the reverse direction out of coil HC and into the diode array detector.

**Figure 4:**
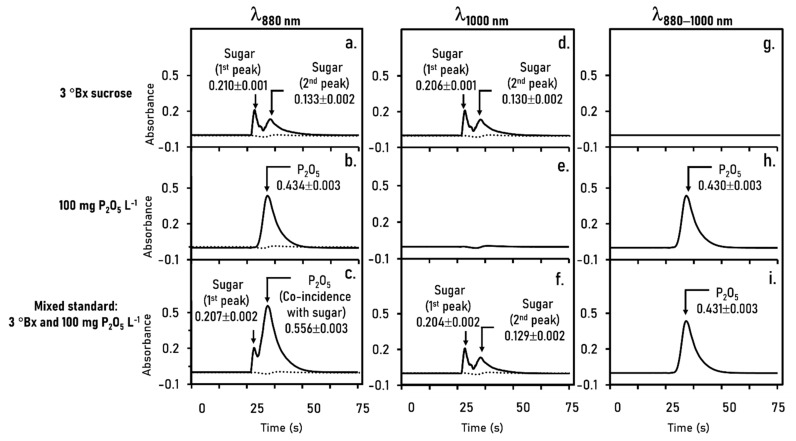
Compensation of the schlieren effect for orthophosphate analysis. The flow profiles obtained when) standard solution of 3 °Bx sucrose (**a**,**d**,**g**), 100 mg P_2_O_5_ L^−1^ (**b**,**e**,**h**) and mixed standard (**c**,**f**,**i**) as plug S in zone sequence in Figure 1c, operated by the SIA system in Figure 1a. A_880_: absorbance at analytical wavelength; A_1000_: absorbance at reference wavelength; A_880__−1000_: corrected absorbance. Dashed line indicating the reagent blank signal. Numbers presenting the mean ± standard deviation of absorbance values obtained from triplicate cycle runs.

We initially monitored the absorbance signal at wavelength of 880 nm which is a maximum absorption wavelength of the molybdenum blue complex. Pure water was used as blank, showing only a small disturbance in the baseline signal (see dashed lines in Figure 4a–c). Figure 4a shows the absorbance profile when 3 °Bx sucrose was the plug S, two Schlieren peaks are clearly seen. The rise of the first and the second Schlieren peaks are due to the passage of the head and the tail through the detection cell. Figure 4b shows the profile obtained when slug S is 100 mg P_2_O_5_ L^−1^ solution. An absorbance peak of the molybdenum blue complex is observed. Figure 4c shows the profile when plug S is a solution of sucrose and phosphate. The first peak in the profile of Figure 4a (0.207 ± 0.002 abs unit) is the Schlieren signal due to sucrose, with an amplitude comparable to the first Schlieren peak in Figure 4b (0.210 ± 0.001 abs unit). However, the second peak in Figure 2c comprises the signal from schlieren effect of sucrose and the absorbance of the molybdenum blue complex. The difference of the second peak heights in Figure 4b,c (Δh = 0.122 abs unit) is approximately equal to the height of the second schlieren peak of Figure 4a (0.133 abs unit). Thus, the absorbance peak for the molybdenum blue complex also contains the schlieren band of the sucrose component. Therefore, the detection wavelength of 880 nm is not suitable since it would cause an error in quantification of orthophosphate in sugarcane juice.

To overcome the Schlieren effect on quantification of orthophosphate, a concept of dual-wavelength spectrophotometry was exploited to compensate the Schlieren effect with subtraction of non-specific absorbance from sucrose. We, therefore, selected the wavelength of 1000 nm as a reference wavelength, where the molybdenum blue complex does not absorb the light. As seen in Figure 4e, there is no light absorption of the blue complex. In contrast, the schlieren profiles of sucrose were observed for pure standard (Figure 4d) and mixed standard (Figure 4f) due to the light deflection. Corrected absorbance was carried out by subtracting the absorbance profile at 880 nm by the absorbance profile at 1000 nm. Figure 4g,h shows the corrected flow profiles for pure sucrose and pure phosphate, respectively. For mixed standard of sucrose with phosphate, the absorbance profile in Figure 4i shows a single peak of corrected phosphate signal. The analytical signal measured for pure phosphate solution (Figure 4b: 0.434 ± 0.003) was in good agreement with the corrected signal for mixed standard (Figure 4i: 0.437 ± 0.003). This confirms that the compensation approach, based on use of dual-wavelength spectrophotometry, is applicable to overcome the schlieren effect in our flow-based analysis. In addition, dual-wavelength measurement offers an advantage in term of usability of a set of mixed standard solutions of sucrose and phosphate calibration graphs. Sensitivities obtained from mixed standards were comparable to those from pure standards.

In practical, detection wavelength of 1000 nm was used for analysis of °Bx, whereas analysis of orthophosphate is carried out by dual-wavelength measurement at 880 and 1000 nm.

### 2.5. System Optimization

#### 2.5.1. Sample Volume

In this work, the volume of sample S in zone sequence for pH measurement (Figure 1c) was not investigated. We observed that a minimum of sample at 1500 μL was enough to fill in the PTFE flow line and the empty chamber of ISFET flow cell (600 μL).

For analysis of °Bx and orthophosphate, the volume of sample S in zone sequence in Figure 1c was investigated to give the required sensitivity with suitable linear ranges. The data in Table 2 show that increasing the volume size of plug S resulted in better sensitivity. However, increasing the volume above a certain value led to significant reduction in linearity range. As s compromise between high sensitivity and wide working range, volume of 50 μL was chosen as the optimum volume of the plug S for analysis of °Bx (by light deflection of Schlieren effect) and orthophosphate (by light absorption of the molybdenum blue complex).

#### 2.5.2. Reagent Concentrations for Orthophosphate Analysis

**Figure 5:**
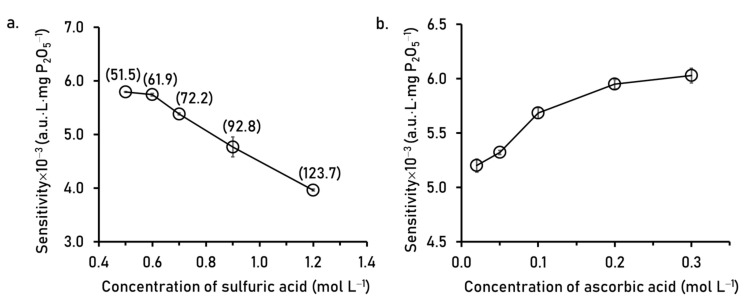
Effect of concentrations of (**a**) sulfuric acid in R1 and (**b**) ascorbic acid in R2 on the sensitivity for phosphate analysis. The numbers in parenthesis are the ratio of [H^+^]:[MoO_4_^2−^].

It is recommended in the literature [28] that the ratio of [H^+^]:[MoO_4_^2−^] should be from 45 to 80 (pH 0.36–1.06) for complete color formation of the molybdenum blue with no self-reduction of molybdate ion. The concentration of sulfuric acid in R1 was varied with the concentration of molybdate ion fixed at 9.7 mmol L^−1^. The results in Figure 5a show that sensitivity decreased when the acid concentration increased due to the increasing of unreactive phosphate at higher ratio of [H^+^]:[MoO_4_^2−^] than the recommended values. For this work, the concentration sulfuric acid in R1 was set at 0.6 mol L^−1^, giving the ratio of [H^+^]:[MoO_4_^2−^] of 61.9.

Ascorbic acid is the reducing agent in R2 and its concentration was varied from 0.02 to 0.3 mol L^−1^. The results in Figure 5b show that sensitivity improved when increasing the concentration of ascorbic acid from 0.02 to 0.1 mol L^−1^. However, sensitivity was not much increased when concentrations of the reducing agent were above 0.1 mol L^−1^. The concentration of 0.2 mol L^−1^was selected for the final operating condition.

### 2.6. Interference Study

Regarding the sample pretreatment with fourfold dilution, tolerance level of interference species in this study represents in the diluted sample.

**Figure 6:**
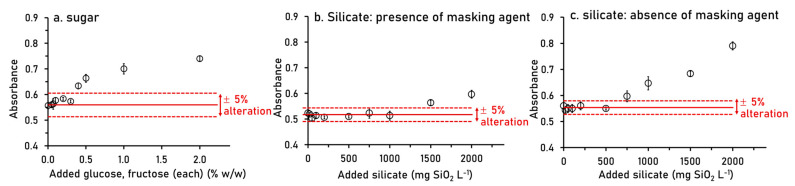
Results from the study for tolerance limits of the developed SIA system for (**a**) glucose and fructose on 3 °Bx sucrose and for silicate on 100 mg P_2_O_5_ L^−1^, (**b**) with tartaric acid masking and (**c**) without masking agent. The red solid lines mean signal of mixed standard of 3 °Bx sucrose and 100 mg P_2_O_5_ L^−1^. The red dashed lines mean the tolerance limit of ± 5% alteration.

Besides sucrose, sugarcane juice also contains glucose and fructose but at much lower amounts (glucose at 0.28–0.98% (*w*/*w*) and fructose at 0.31–0.70% (*w*/*w*) [2]. Therefore, the estimated concentration of each of these monosaccharide sugar in the diluted sample would be, at the maximum, 0.25% (*w*/*w*). According to the result in Figure 6a, it was found that our method can tolerate each sugar up to 0.3% (*w*/*w*), which is higher than the maximum concentration found in diluted sample.

In the analysis of orthophosphate, silicate is the most common interference when the molybdenum blue method is employed [29]. In sugarcane juice, the concentration of silicate can be twice that of phosphate [2], in a range of 600–800 mg SiO_2_ L^−1^). The tolerance levels of our method in presence and absence of masking agent (0.016 mol L^−1^ tartaric acid) in R1 [30] were investigated. As the result in Figure 6b, addition of tartaric acid increases the tolerance level up to 1000 mg SiO_2_ L^−1^ (for diluted sample), hence indicating the tolerance level are up to 4000 mg SiO_2_ L^−1^ (for undiluted sample). We also found that absence of masking agent gave the tolerance level up to 500 mg SiO_2_ L^−1^ for fourfold diluted sample (see Figure 6c), in which tolerate silicate up to 2000 mg SiO_2_ L^−1^ for undiluted sample. Even though both investigations gave tolerance levels much greater than silicate concentrations normally found in sugarcane juice; however, we chose to add tartaric masking agent in R1 to ensure that silicate would not interfere the analysis of orthophosphate.

### 2.7. Analytical Features and Application

The developed SIA system in Figure 1a with zone sequences in Figure 1b,c provides for simultaneous quantitative analyses of pH, sucrose and orthophosphate in raw sugarcane juice. Using the optimal conditions, analytical features are summarized in Table 3. Working ranges of these key parameters are fit to the purpose for analysis in raw sugarcane juice. Limit of detection and repeatability are satisfied with reasonable throughput of 12 injections per hour for simultaneous analysis of pH, sucrose and orthophosphate. 

The developed flow system was applied to nine samples of sugarcane juice (Table 4). Using the paired *t*-test [31], the results obtained from this system were not significantly different to the results obtained using the comparative methods (pH: *t*_observe_ = 0.57, *t*_critical_ = 2.31, *P* = 0.05; sucrose: *t*_observe_ = 0.39, *t*_critical_ = 2.31, *P* = 0.05; phosphate: *t*_observe_ = 0.71, *t*_critical_= 2.31, *P* = 0.05). Pearson’s correlations also confirmed that our SIA system gave results that did not differ significantly from values using the comparative methods (y = 1.002x + 0.083, r^2^ = 0.999). The percent recoveries of spiked standard (2 °Bx sucrose and 50 mg P_2_O_5_ L^−1^) in nine samples were in ranges of 84–105% for sucrose and 87–122% for orthophosphate. In this work, recoveries for sucrose and orthophosphate were acceptable since they were from entire analytical procedure, including step of sample preparation (dilution, color removal, centrifugation and filtration) and step of analysis by our flow method.

### 2.8. Comparison with Other Flow-Based Methods for Analysis of Sugarcane Juice

Our proposed SIA manifold is the first development for simultaneous analysis of °Bx, orthophosphate and pH in sugarcane juice. However, there are some flow-based systems designed for analysis of sugars with various detection modes. Table 5 lists the analytical features for the determination of sugar, i.e., glucose, sucrose and total reducing sugars, obtained from our SIA manifold as compared with other flow-based systems.

Methods A–D, employing FIA and multi-commutation system, are based on the spectrophotometric detection. Light absorption was applied to detect the product after sugar was chemically reacted with oxidizing agent [8,9,10] or enzyme [13] with well-known chemical reactions. Method E employs the FIA-gravimetric detection by online weighing of filtered copper(I) oxide precipitates after total reducing sugar was oxidized by Fehling reagent [11]. Method F utilizes the FIA-piezoelectric detection [12] based on measurement of change in frequency of the detector with variation of viscosity and density of solution. Figures of merit achieved from Methods A to F were sensitive an gave a rapid measurement (20–70 sample h^−1^).

Regarding our developed SIA (Method G), sucrose analysis is reagent-free method. Our SIA method also offers simultaneous analysis of sucrose, orthophosphate and pH within 6 min per analysis cycle (10 sample h^−1^, see operational steps in Table 5). A slug of sample (20 μL) was served to dual detection of sucrose and orthophosphate. Significant reduction in consumption of the reagents (40 μL) for molybdenum blue method was achieved. Analytical figures of merit, i.e., working ranges, LODs and precisions, for three parameters were reasonably acceptable to fit the purpose.

In addition, Method G with use of the light absorption of molybdenum blue complex offers advantages over our previous report [19] using in-line turbidimetric detection of calcium phosphate particles, i.e., lower detection limit (light absorption: 0.59 mgP L^−1^; turbidimetry: 6.53 mgP L^−1^) and no need of washing step for accumulative adsorption of calcium phosphate precipitates on the inner wall of Teflon tubing.

## 3. Materials and Methods

### 3.1. Chemicals and Reagents

All solutions were prepared in deionized (DI) Milli-Q^®^ water. The sucrose standard used in this work was commercial grade sugar (Mitr Phol, Chaiyaphum, Thailand). A 50 °Bx stock sucrose solution was prepared by dissolving exactly 50.00 g of solid sucrose in 50.00 g of DI water, with stirring on a magnetic stirrer until the solid has completely dissolved. The stock solution was stored at 4 °C and used within one week.

A 1000 mg P_2_O_5_ L^−1^ stock phosphate solution was prepared by exactly dissolving 0.2 g of disodium hydrogenphosphate (Fluka, Buchs, Switzerland) in 100.0 mL DI water. Disodium hydrogenphosphate was previously dried at 60 °C for 2 h and stored in a desiccator.

Working standard solutions used for calibration were mixed standard solutions of sucrose and phosphate, prepared by appropriate dilution with DI water of the stock solutions.

Acidic molybdate solution (9.7 mmol L^−1^ molybdate in 0.6 mol L^−1^ sulfuric acid was prepared by dissolving 6.0 g of ammonium heptamolybdatetetrahydrate (QRëC, Auckland, New Zealand) in 300 mL of DI water. Then, approximately 16.0 mL of concentrated sulfuric acid (RCI Labscan, Dublin, Ireland) was slowly added and the final solution diluted to 500 mL with DI water (Reagent R1). L-ascorbic acid solution at 0.2 mol L^−1^ was prepared by dissolving 3.5 g of L-ascorbic acid (Fisher, Leicestershire, UK) in 100 mL of DI water (Reagent R2).

Buffer solutions with pH 4.01, 7.00 and 10.00 (Thermo Scientific™, Beverly, MA, USA) were used to calibrate the ISFET sensor by measuring the voltage output. 

### 3.2. Sugarcane Juice Sample

Samples of the raw sugarcane juice (S1–S9) were prepared by crushing fresh sugarcane stalks, purchased from nine different local markets in Bangkok, Thailand. The sugarcane juice was diluted fourfold with water. Then, 0.2 g of fine activated charcoal powder (particle size ≤ 40 μm, Fluka, Brussels, Belgium) was added to 20.0 mL aliquot of the diluted sugarcane juice to remove colored compounds. The sample was then centrifuged at 3000 rpm for 20 min to separate the charcoal powder and solid debris. The supernatant was filtered through a 0.45 μm cellulose acetate membrane filter (Sartorius, Hanover, Germany) before analysis of sucrose, orthophosphate and pH using the developed SIA system.

Recovery study was carried out by spiking a 3.00 mL of the 50 °Bx stock sucrose solution and a 2.50 mL of the 1000 mg P_2_O_5_ L^−1^ stock phosphate solution into 100.0-mL volumetric flask containing 25.00 mL of sugarcane sample, prior making up volume to 100.0 mL with DI water. Percentages of recoveries were determined by the following Equation (1):

(1) $$\%\mathrm{Recovery}=\left(\frac{C_{\mathrm{spiked}\;\mathrm{sample}}-C_{\mathrm{sample}}\;}{C_{\mathrm{standard}}}\right)\times 100$$

where C_spiked sample_: concentration of °Bx or P_2_O_5_ found in sample spiked with standard; C_sample_: concentration of °Bx or P_2_O_5_ found in sample and C_standard_: concentration of standard.

### 3.3. The SIA System

As shown in Figure 1a, the SIA system consisting of a syringe pump, SP (PSD/4 Hamilton, Reno, NV, USA) with an 8-port selection valve, SV (MVP Hamilton, Reno, NV, USA) was used to control the carrier and reagent flows. PTFE tubes with 1.0 mm and 1.59 mm i.d. (Cole Parmer, Waltham, MA, USA) were used for the flow path and holding coil, HC, respectively. The MGC-MPV LMPro (version 5.2) software was used for controlling the syringe pump and the selection valve of the SIA system. Zone sequences for pH and for Brix and orthophosphate are depicted in Figure 1b,c. The overall flow protocols for the analysis of raw sugarcane juice are given in Table 1.

An ISFET sensor (Winsense Co., Ltd., Bangkok, Thailand) consisting of a flow-through cell (Figure 2a) and a power supply circuit (Figure 2b) and was used to measure the pH. In-house software was written using LabVIEW 8.0^TM^ to record data and store on a computer via RS-232 port. An Agilent photodiode array UV–visible spectrophotometer (Model 8453, Waldbronn, Germany), equipped with a regular 10 mm light path flow cell (Hellma, Waldbronn, Germany), was used for monitoring the absorbance at 880 and 1000 nm for analysis of sucrose and orthophosphate.

## 4. Conclusions

This work presents the development of an automated flow system for simultaneous analysis of degree Brix (°Bx), orthophosphate and pH in raw sugarcane juice. The sequential injection (SI) format was selected for precision and automation of liquid handling at micro liter level. Analysis of °Bx, as sucrose content, was carried out by detecting the light refraction at interface of sucrose and water (the so-called Schlieren effect), whereas orthophosphate determination was carried out by detecting the absorbance of molybdenum blue complex. Only one spectrophotometer detector set at 880 and 1000 nm was required to measure both analytes, leading to achievement in compensation of the schlieren effect from sucrose for the determination of phosphate by using a dual-wavelength detection. Analytical signal of sucrose was based on the wavelength of 1000 nm, whereas analytical signal of phosphate was a signal subtraction between 880 and 1000 nm. Analysis of pH was carried out by using the ion-selective field-effect transistor (ISFET) as a flow-through detection cell. The ISFET signal rises accordingly to the H^+^ concentration.

With the optimized flow configuration and zone stacking sequences, simultaneous analysis of sucrose, orthophosphate and pH was accomplished within 5.5 min. This SIA system significantly reduced consumption of reagents (40 μL) compared to previous reports (mL level) for orthophosphate measurement in sugarcane juice based on formation of molybdenum blue complex [16]. Applications to raw sugarcane samples gave statistical agreement with comparative methods. The developed system is simple and suitable for routine use in the cane sugar industry.

## Figures and Tables

**Table 1:** Operational step for the SIA system shown in Figure 1 for analysis of pH, °Bx and orthophosphate in sugarcane juice.

Step	Syringe Valve Port	Motion of SP	Connection of SV Port	Flow Rate (μL s^−1^)	Volume (μL)	Description	Period (s)
pH Measurement							
1	Out	Down	HC-SV-P8	10	25	Air loaded into HC	2.5
2	Out	Down	HC-SV-P6	50	1500	Sample (S) loaded into HC	30
3	Out	Down	HC-SV-P8	10	25	Air loaded into HC	2.5
4	Out	Up	HC-SV-P1	50	1550	Sample in HC moved toward the ISFET detector and waste (W3) for pH measurement	31
Clean the Flow Path of HC							
5	In	Down	-	100	5000	Water carrier loaded into SP	50
6	Out	Up	HC-SV-P5	100	5000	Water carrier dispensed to waste (W1) for cleaning the flow path	50
Degree Brix and Orthophosphate Measurements							
7	In	Down	-	100	2500	Water carrier loaded into SP	25
8	Out	Down	HC-SV-P4	20	100	Ascorbic acid solution (R2) loaded into HC	5
9	Out	Down	HC-SV-P3	20	100	Acidic molybdate solution (R1) loaded into HC	5
10	Out	Down	HC-SV-P6	20	50	Sample (S) loaded into HC	2.5
11	Out	Up	HC-SV-P2	100	2750	Solutions in HC transferred to the diode-array detector and waste (W2) for recording of the flow profile	27.5
Clean the Flow Path to ISFET Detector							
12	In	Down	-	100	5000	Water carrier loaded into SP	50
13	Out	Up	HC-SV-P1	100	5000	Water carrier in SP moved toward the ISFET detector and waste (W3)	50

Total analysis time is 331 s per cycle (~10 sample per h).

**Table 2:** Effect of sample volume for the analysis of degree Brix and orthophosphate in sugarcane juice.

Sample Volume (μL)	Degree Brix		Orthophosphate	
	Calibration Equation, r^2^	Linearity Range (°Bx)	Calibration Equation, r^2^	Linearity Range (mg P_2_O_5_ L^−1^)
25	y = (3.35 × 10^−2^)x + (0.28 × 10^−2^), r^2^ = 0.999	1.0–7.0	y = (3.04 × 10^−3^)x + (3.39 × 10^−2^), r^2^ = 0.999	20–200
50	y = (5.71 × 10^−2^)x + (0.39 × 10^−2^), r^2^ = 0.999	1.0–7.0	y = (5.62 × 10^−3^)x + (3.36 × 10^−2^), r^2^ = 0.999	20–200
75	y = (8.50 × 10^−2^)x + (0.31 × 10^−2^), r^2^ = 0.995	1.0–5.0	y = (12.3 × 10^−3^)x + (2.41 × 10^−2^), r^2^ = 0.999	20–200
100	y = (8.41 × 10^−2^)x + (0.18 × 10^−2^), r^2^ = 0.993	1.0–5.0	y = (18.0 × 10^−3^)x + (4.11 × 10^−2^), r^2^ = 0.996	20–150

y: Abs_1000 nm_ and Abs_880_−_1000 nm_, for analysis of sugar and orthophosphate, respectively. x: °Bx and mg P_2_O_5_ L^−1^, for analysis of sugar and orthophosphate, respectively. r^2^: Coefficient of determination.

**Table 3:** Analytical features of the SIA system (Figure 1a) for measurements of pH, degree Brix and orthophosphate in raw sugarcane juice.

Feature	Performance		
	pH	Degree Brix	Orthophosphate
Working range	0–14 pH	1.0–7.0 °Bx sucrose	20–200 mg P_2_O_5_ L^−1^
Example calibration equation	mV = (59.17 ± 0.48) pH + (434.2 ± 3.6)	Abs_1000 nm_ = [(5.71 ± 0.12) × 10^−3^] °Bx + (0.39 ± 0.34) × 10^−2^	Abs_880-1000 nm_ = [(5.62 ± 0.22) × 10^−3^] mg P_2_O_5_ L^−1^ + (3.36 ± 1.01) × 10^−2^
Coefficient of determination (r^2^)	0.999	0.999	0.994
Limit of Detection (LOD)	-	0.2 ^a^	5.4 ^a^
Repeatability (% RSD)	2.9 ^b^, 2.5 ^c^, 2.4 ^d^	3.0 ^e^	2.7 ^e^
Throughput (injection h^−1^)	12		

^a^ defined as 3SD of intercept/slope. ^b,c,d^ obtained from ten replicate injections of standard buffer solution pH 4.01 ^b^, 7.00 ^c^ and 10.01 ^d^, respectively. ^e^ obtained from ten replicate injections of mixed standard solution of 3 °Bx sucrose and 100 mg P_2_O_5_ L^−1^.

**Table 4:** Analysis of sugarcane juice using the developed SIA (Figure 1a) system as compared with comparative methods. Quantification was carried out in triplicate measurements.

Sample	pH		Degree Brix		Orthophosphate (mg P_2_O_5_ L^−1^) ^a^	
	SIA—ISFET	Conventional pH Glass Electrode ^b^	SIA—Schlieren Method	Refractometry ^c^	SIA	Batchwise ^d^
S1	5.23 ± 0.08	5.11 ± 0.15	14.4 ± 0.8	14.0 ± 0.3	311 ± 14	308 ± 11
S2	5.12 ± 0.10	5.42 ± 0.18	14.9 ± 0.8	15.2 ± 0.7	321 ± 14	317 ± 6
S3	5.37 ± 0.24	5.36 ± 0.18	14.0 ± 0.6	13.8 ± 0.3	309 ± 14	308 ± 10
S4	5.41 ± 0.12	5.50 ± 0.12	13.3 ± 0.1	13.8 ± 0.6	359 ± 18	358 ± 14
S5	5.32 ± 0.14	5.21 ± 0.14	13.7 ± 0.2	14.6 ± 0.7	366 ± 12	362 ± 11
S6	5.13 ± 0.11	5.19 ± 0.21	14.3 ± 0.1	13.9 ± 0.6	352 ± 14	347 ± 12
S7	5.48 ± 0.21	5.12 ± 0.10	14.3 ± 0.1	14.0 ± 0.3	357 ± 18	356 ± 18
S8	5.30 ± 0.16	5.19 ± 0.15	14.1 ± 0.2	13.7 ± 0.6	340 ± 10	341 ± 16
S9	5.20 ± 0.14	5.15 ± 0.18	15.4 ± 0.2	14.8 ± 0.1	359 ± 14	368 ± 8

^a^ Based on the molybdenum blue method. ^b^ pH glass electrode Routine 51,343,050 and pH meter FiveEasy Plus™ FEP20, Mettler Toledo, Toledo, OH, USA. ^c^ Refractometer 30PX/GS, Mettler Toledo, Toledo, OH, USA. ^d^ ICUMSA method [32].

**Table 5:** Comparison of reported analytical flow-based methods for analysis of °Bx in sugarcane juice.

Method/Analyte	Detection	Volume (μL) (Sample: V_S;_ Reagent: V_R)_	Working Range	LOD	Precision (as RSD)	Throughput (h^−1^)
A. Spectrophotometric FIA [9]/ Sucrose	Light absorption at 350 nm of I_3_^−^ after sample chemically reacting with IO_4_^−^ and I^−^.	V_S_: NA V_R_: NA	0.025–0.20% (*w*/*v*)	NA	0.51% ^d^	30
B. Spectrophotometric FIA [8]/ Sucrose and total reducing sugars ^a^	Light absorption (512 nm) after sample oxidized by Fe(CN)_6_^3+^ and produced Fe(CN)_6_^2+^ mixture of 1–10 phenanthroline and Fe^3+^, respectively.	V_S_: NA V_R_: NA	0.001–0.020% (*w*/*v*)	NA	0.38% ^d^ (sucrose) 0.47% ^d^ (total reducing sugars)	40
C. Spectrophotometric FIA [10]/ Total reducing sugars ^a^	Light absorption (410 nm) after sample oxidized by Fe(CN)_6_^3+^ in alkaline condition, with assisting a focalized coiled reactor in a microwave oven.	V_S_: 50 V_R_: cont. flow ^b^	0.0009–0.0216% (*w*/*v*)	0.00027% (*w*/*v*)	< 1.4% ^f^	70
D. Spectrophotometric-Multi-commutation [13]/ Glucose	Light absorption (510 nm) after sample reacting with glucose-oxidase (GOD) enzyme and mixture of 4-aminophenazone and phenol, respectively.	V_S_: 5.6 V_R_: 16.8	0.05–0.2% (*w*/*v*)	NA	0.12% ^d^, 0.3% ^g^	30
E. FIA-gravimetric [11]/ Total reducing sugars ^a^	Weighing of filtered precipitate Cu_2_O.	V_S_: NA, V_R_: NA	0.2–1.0% (*w*/*v*)	NA	0.9% ^e^	20
F. FIA-piezoelectric [12]/ Sucrose	Changing in frequency of piezoelectric detector due to the density of sucrose solution.	V_S_: 150, V_R_: NR	0.5–20% (*w*/*v*)	0.3% (*w*/*v*)	< 1.45% ^h^	70
G. SIA (This work)						
Sucrose	Schlieren effect	V_S_: 20 ^c^, V_R_: NR	0.5–5 °Bx	0.2	3.2% ^i^	10
Orthophosphate	Molybdenum blue method	V_S_: 20 ^c^, V_R_: 40	20–200 mg L^−1^ P_2_O_5_	5.4	2.7% ^i^	
pH	ISFET	V_S_: 1500, V_R_: NR	0–14 pH	NA	2.9 ^j1^, 2.5 ^j2^, 2.4 ^j3^	

NA: not available. NR: not required. ^a^ Total reducing sugars: mixture of glucose and fructose. ^b^ Cont. flow: continuous flow of reagent stream. ^c^ Same sample plug used for analysis of sucrose and phosphate. ^d^ Average of variation coefficient from 7 samples and ^e^ from 8 samples. ^f^ Ten injections of 0.0072% and 0.0144% standard. ^g^ Six injections of 0.1% (*w*/*v*) standard. ^h^ Fifteen injections of 6% (*w*/*v*) standard. ^i^ Ten injection of mixed standard of sucrose and 100 mg P_2_O_5_ L^−1^. ^j^ Ten injections of standard buffer solution pH 4.01 ^j1^, 7.00 ^j2^ and 10.01 ^j3^.

## Data Availability

The data presented in this study are available on request from the corresponding authors.

## Sample Availability

Samples of the compounds are available from the authors.
